# Integration of community home based care programmes within national primary health care revitalisation strategies in Ethiopia, Malawi, South-Africa and Zambia: a comparative assessment

**DOI:** 10.1186/s12992-014-0085-5

**Published:** 2014-12-11

**Authors:** Carolien Aantjes, Tim Quinlan, Joske Bunders

**Affiliations:** Faculty of Earth and Life Sciences: Athena Institute, VU University Amsterdam, De Boelelaan 1085, 1081 HV Amsterdam, The Netherlands; ETC. Foundation, Kastanjelaan 5, Leusden, The Netherlands; Health Economics and HIV/AIDS Research Division, University of KwaZulu-Natal, Westville Campus, University Road, Durban, South-Africa

**Keywords:** Community and home based care, Primary health care, Integration, Co-ordination, Integrated care, Chronic care

## Abstract

**Background:**

In 2008, the WHO facilitated the primary health care (PHC) revitalisation agenda. The purpose was to strengthen African health systems in order to address communicable and non-communicable diseases. Our aim was to assess the position of civil society-led community home based care programmes (CHBC), which serve the needs of patients with HIV, within this agenda. We examined how their roles and place in health systems evolved, and the prospects for these programmes in national policies and strategies to revitalise PHC, as new health care demands arise.

**Methods:**

The study was conducted in Ethiopia, Malawi, South Africa and Zambia and used an historical, comparative research design. We used purposive sampling in the selection of countries and case studies of CHBC programmes. Qualitative methods included semi-structured interviews, focus group discussions, service observation and community mapping exercises. Quantitative methods included questionnaire surveys.

**Results:**

The capacity of PHC services increased rapidly in the mid-to-late 2000s via CHBC programme facilitation of community mobilisation and participation in primary care services and the exceptional investments for HIV/AIDS. CHBC programmes diversified their services in response to the changing health and social care needs of patients on lifelong anti-retroviral therapy and there is a general trend to extend service delivery beyond HIV-infected patients. We observed similarities in the way the governments of South Africa, Malawi and Zambia are integrating CHBC programmes into PHC by making PHC facilities the focal point for management and state-paid community health workers responsible for the supervision of community-based activities. Contextual differences were found between Ethiopia, South Africa, Malawi and Zambia, whereby the policy direction of the latter two countries is to have in place structures and mechanisms that actively connect health and social welfare interventions from governmental and non-governmental actors.

**Conclusions:**

Countries may differ in the means to integrate and co-ordinate government and civil society agencies but the net result is expanded PHC capacity. In a context of changing health care demands, CHBC programmes are a vital mechanism for the delivery of primary health and social welfare services.

## Background

This article describes Primary Health Care (PHC) revitalisation strategies in four African countries on the basis of co-ordination of efforts between government and civil society and the integration of civil society-led community home based care (CHBC) services into PHC service structures. We studied these processes, using HIV-focused CHBC programmes as our point of reference. The research rationale was the general lack of empirical data on the place and role of CHBC in African health systems in a context of changing health care demands and international health strategies.

The period post Alma Ata can be described as a turbulent time for African health systems. Major events, such as the HIV/AIDS epidemic, the structural adjustment programmes, development of global health initiatives and funding for priority diseases have had a profound impact on the way health systems deliver PHC [1,2]. The emergence of chronic, non-communicable diseases (NCDs) and changes in the demographic composition of populations are more recent influences [3]. In 2008, the WHO drove a PHC ‘revitalisation’ agenda via a conference with African health ministers; the outcome of which was the Ouagadougou Declaration which espoused commitment to the PHC principles formulated at Alma Ata in 1978 alongside commitment to current international health agenda [4]. To illustrate, the Declaration calls on the signatories:

“to update their national health policies and plans according to the Primary Health Care approach, with a view to strengthening health systems in order to achieve the Millennium Development Goals, specifically those related to communicable and non-communicable diseases, including HIV/AIDS, tuberculosis and malaria; child health; maternal health; trauma; and the emerging burden of chronic diseases” ([5] p.13).

We framed our research questions from an historic perspective to assess changes in the provision of CHBC following the introduction of anti-retroviral treatment and changes in the level of government involvement in CHBC. CHBC programmes are an elaboration of ‘home-based’ care (HBC) that focused on palliative care prior to the advent of ART programmes for patients with HIV/AIDS [6-12]. During the 1980s, there was a proliferation of community health workers (CHW) in the guise of lay volunteers recruited and deployed by non-governmental organisations (NGOs) and faith-based organisations (FBOs) who provided home based care to the many people who succumbed to the disease. Throughout the 1990s, these initiatives grew with international funding support for pragmatic reasons: HBC helped to mitigate the shortage of medical personnel [9,13,14]. That rationale supported further expansion of HBC programmes following the introduction of ART in many African countries in the mid-2000s. A key development was diversification of HBC services to support the patient recruitment to ART programmes, patient adherence to medicinal regimens and retention on treatment [15-18]. Service diversification also involved increased community participation to enable provision of support to ART patients to rebuild their lives and livelihoods and to assist staff in PHC facilities; hence, the gradual shift in terminology from HBC to CHBC [9,19].

Together, ART and CHBC programmes form a foundation for the elaboration of primary care services which are capable of confronting the new demands on health systems such as the need for chronic care services in the face of the anticipated increase in NCDs in Africa [20-23]. Accordingly, PHC services are pivotal in the provision of comprehensive care and a continuum of care [24-26] for patients who have recurrent engagements with the health system, as a result of need for long term, even lifelong treatment as is the case for HIV patients, age-related illnesses and various NCDs [27]. Although PHC facilities are their regular point of access to health care [28], most of their care takes place outside the facility and will continue to do so [29]. To illustrate, the WHO model for chronic care states that ‘community partners’, patients and family members in the provision of chronic care are as essential as health professionals [30].

Inevitably, this logic reiterates integration and co-ordination as fundamental principles of PHC. To illustrate, the Ouagadougou Declaration explicitly refers to these principles. On co-ordination, the framework proposed development of:

“Mechanisms to involve all private health providers to ensure a continuum of care among all citizens, regardless of their economic status” and, strengthening of “co-ordination and collaboration with civil society organisations, particularly CBOs and NGOs, in community health development” [31].

On integration, the framework proposed development of:

“Integrated service delivery models at all levels, taking into account the referral system regardless of the organisation and nature of the services (promotive, preventive, curative and rehabilitative) so as to improve the economic efficiency and equity of health services delivery” [31].

It should be noted that the framework reiterated WHO and UNAIDS advocacy of these principles [32-35] and longstanding emphasis of the principles in research literature [24,36-42]. Furthermore, the definitions in the framework summarise theoretical discussions and debates on the different forms and degrees of co-ordination and integration in the research literature [43].

One focus of our research was on how these principles are being applied in Malawi, Zambia, South Africa and Ethiopia. That is the focus of this paper, in line with the general empirical purpose of the study, and with particular reference to different and common practical means by which governments in those countries are attempting to co-ordinate and integrate government and civil society health programmes.

## Methods

We draw on results from an historical comparative study of CHBC programmes conducted in Malawi, Zambia, South Africa and Ethiopia in 2011 and 2012 [15,44-47]. It involved purposive sampling of countries; criteria for selection included government commitment to the PHC revitalisation agenda, generalised HIV epidemic, and well established (10 > years old) HBC/CHBC programmes. There were four research objectives:

1. Explore the adaptations and changes in caregiving at the community level since the rapid scale up of anti-retroviral therapy while focusing on the tasks of caregivers and the needs of their clients;
2. Assess how and to what extent caregiving by informal caregivers at community level has been integrated in the health system and is being recognised as part of primary health care structures and policies;
3. Investigate the contributions, potential role of and benefits for caregivers in the expansion of HIV prevention, treatment and primary health care programmes;
4. Assess the potential means for formal and informal community health worker programmes to complement each other in the context of decentralisation of HIV treatment programmes, taking into account current initiatives and arrangements.

An historical perspective was used to gain insight into the changing form and content of CHBC and PHC in Africa. A comparative approach, borrowed from the discipline of social anthropology and longstanding methodology in that discipline, was used for being a strong methodological means for qualitative studies to distinguish commonalities and differences of phenomena in different settings. Our interest was to identify, from a number of case studies, common patterns and trends in the evolution and integration of CHBC in African health systems. We used a variety of methods, both qualitative and quantitative, and included a wide range of informants in order to obtain a comprehensive insight into the research objectives. Table 1 summarises the methods, sample categories and sizes. We commenced with an online survey to obtain the views of international experts on HIV and PHC with regard to the proposed research and its foci. The results of that survey informed the development of our research tools. Local research teams conducted the field research, having previously assisted with standardisation of the research tools across the four countries. The first part of the field research consisted of a desk review and a round of semi-structured interviews with key informants at national level, in which the four research objectives were addressed via an exploration on the evolution in community care and PHC policy and practices, perspectives on CHBC, in-country community care structures and models, and linkages and networks between governmental and non-governmental organisations. Key informants included officials from Ministries of Health, Ministries of Community Development and/or Welfare, national AIDS coordinating bodies, large care and support organisations, HIV patient networks, and funders of CHBC programmes.

**Table 1:** **Study sample**

**Research method and sample categories**	**Sample size**
Online survey among international experts	17
Key national level informants in government and care organisations	49
Key informants CHBC programmes	71
FGD with CHBC programme staff	17
FGD with secondary caregivers in the CHBC programmes	115
FGD with community representatives in the CHBC programmes	65
Individual interviews with clients	98
Individual interviews with primary caregivers	99
Validation Interviews with key informants at national level	21
Validation Questionnaire survey among care and support organisations	46

The research then continued with in-depth case studies of three CHBC programmes, led by civil society organisations (CSOs), in each country. The sampling criteria were that the selected programmes a) had been operational for more than 10 years, b) were managed by different organisations, c) were generally representative of CHBC programmes in each country, d) offered diversity of services in care (not exclusively health) as well as in their range of clients (HIV as well as other chronic illnesses), and e) that the sample included both urban and rural programmes. In order to minimise selection and information bias by the researchers, actual selection was facilitated by consultation with ‘advisory boards’ set up in each country for the study. These boards consisted of representatives of CHBC programmes, staff from national HIV programmes and community caregivers. In each programme, semi-structured interviews and timeline exercises were conducted with staff members and external stakeholders (such as local clinic staff), as well as focus group discussions (FGDs) and community mapping exercises with community representatives and caregivers, service observations and structured interviews with patients and their relatives. Semi-structured interviews with programme staff and external stakeholders and FGDs with caregivers and community representatives, among others, explored the position and contribution of CHBC programmes to PHC over time. The timeline method was specifically used to reconstruct the evolution in community care practices before and after the introduction of ART. Community mapping was used to gain insight into the different actors involved in community care and the links between them. The interviews with patients and their relatives retrieved information on the patient needs, the type of support they received and provided within the household and the changes therein following ART.

Repeat interviews with national level informants were conducted in which interim results were discussed. Furthermore, towards the end of the primary research, a questionnaire survey was conducted amongst CHBC service organisations in each country. The short questionnaire was based on the common research results from the study as a whole. It was distributed to 15 care organisations in each country; 12 which had not been involved in the research and three to the managers of the programmes which had participated. The sample organisations in each country were selected in consultation with the country advisory boards, and on the basis of the same programme selection criteria used previously as well as a guideline to include organisations from across each country as well as organisations working in the same region/province where the primary research had been done. Ultimately, 59 questionnaires were distributed and 46 were completed and returned. In addition, the country research reports were presented and discussed with the advisory boards prior to public dissemination.

Data from interviews and focus group discussions were transcribed verbatim, and when FGDs were held in vernacular languages, scripts were translated into English. The analysis of qualitative research data from each country was standardised, using a structured coding model based on the study objectives and recurrent themes in the literature. The coding guide was distributed to local research teams for input and compatibility checks and data subsequently entered into software programme Atlas.ti. Data from the validation questionnaire was processed in SPSS software. The analysis and triangulation of country-level data was carried out by the local research teams. The authors compared and synthesized this data from all four countries, on the basis of detailed country research reports (these were produced at several intervals during the research) and their access to the primary data. Approval for the research was obtained from ethical review boards at the Jimma University, Ethiopia and the University of Cape Town, South Africa, the National Health Sciences Research committee in Malawi, the ERES Converge Ethics Review Board in Zambia and from the VU University Medical Centre, Amsterdam in the Netherlands.

## Results

The four country studies examined two overlapping processes. One, was CSO-driven elaboration of CHBC programmes following the introduction of ART programmes in the mid-2000s and their inter-actions with government health services. The second, was government-led investment in PHC services following the Ouagadougou Declaration. The overlap lies in the general trend within national PHC revitalisation strategies to incorporate CHBC into government PHC services. The first section discusses the evolution of CHBC. We found many similarities across the four countries; hence this is a combined section. The second section discusses the evolution of government involvement in CHBC and the position of these programmes in current PHC revitalisation strategies. This section describes these processes in each country separately as there were notable differences between them. This is followed by a comparative assessment of the four country studies.

### Evolution of CHBC in context of ART

Following the introduction of ART in Ethiopia, Malawi, South Africa and Zambia in 2004/5, CHBC programmes adapted to the changing needs of HIV-infected and affected individuals and families. Data from all four countries emphasised three sets of patient needs: adequate nutrition, cash incomes, and psycho-social support. There was still a need for home-care but, by 2012, there were many patients who had recovered or were recovering their health as a result of ART and, hence, an ever increasing population of ART patients. To illustrate, at the time of this research in 2012, approximately 370,000 (76%) individuals in Malawi were receiving ART out of a total of 480,000 patients eligible for treatment; the respective figures were approximately 450,000 (86%) out of 520,000 individuals in Zambia; 270,000 out of 400,000 (68%) in Ethiopia; and 2 million out of 2.5 million (81%) in South Africa [48].

A feature of CHBC programmes in Ethiopia, Malawi and Zambia were activities to obtain food directly for HIV patients through the charity of the local residents and businesses and/or assistance to starting farming and business ventures. Another significant aspect of patient needs was the growing demand for CHBC programmes to address shortfalls in services as well as new health care challenges; for example, access to anti-retroviral medicines. Expressed concerns of patients were the costs incurred in time and money for transport to collect medicines every month from PHC clinics and/or hospitals. Patients also emphasised that their medical needs were largely for opportunistic illnesses, side effects of treatment and treatment-induced disabilities.

Informants were not simply voicing ‘wish lists’ though, in the case of South Africa, there was a particular demand for services, largely not provided, to address trauma arising from domestic and criminal violence in families and communities and, particularly in urban areas, drug abuse. Otherwise, the needs were stated in contexts where there were already CHBC and government health service interventions that were reference points for adding new services. For example, many CHBC programmes include TB care and support because of high prevalence of TB and HIV co-morbidity. In all four countries, there was a trend amongst CHBC programmes to extend their services beyond HIV to provide, for example, basic nursing care for the elderly and for malaria patients. Table 2 presents the results from our questionnaire survey among a larger sample of CHBC programmes, which gives an indication of the range of activities undertaken by CHBC providers in the delivery of PHC. We observed innovative approaches in the CHBC programmes, such as the establishment of treatment clubs^a^ in South Africa and deployment of Care and Prevention teams^b^ in Zambia, in response to changing health care demands in villages and towns.

**Table 2:** **Diversity of care and support services provided by CHBC organisations**

**Types of care and support**	**Proportion of CHBC organisations providing particular care and support services (%)**			
	**Malawi**	**Zambia**	**Ethiopia**	**South Africa**
Basic HIV/AIDS nursing care	91	78	80	75
Basic nursing care: elderly care	91	67	70	62
Basic nursing care: malaria	54	78	10	6
Basic nursing care: other diseases	82	56	30	69
Clinical care (e.g. taking blood pressure, weighing people) in organisation’s own facility	45	44	20	62
End of life care/palliative care	91	44	50	62
Pain relief	73	78	70	44
Personal hygiene	100	100	80	69
In home/community: pre- ART patient counselling	73	89	100	75
In home/community: ART adherence support	73	89	90	94
In home/community: recruitment of patients for VCT services	73	89	80	87
In home/community: recruitment of patients for ART services	54	89	80	75
In home/community: Recruitment of pregnant women for PMTCT services	18	78	90	87
In home/community: HIV pre-test information	73	100	50	37
In home/community: HIV testing	91	78	0	37
In home/community: HIV post-test counselling	91	89	30	37
Health education: HIV	91	100	80	100
Health education: malaria	54	89	50	6
Health education: other diseases (e.g. TB, STIs)	100	89	60	75
Social support (e.g. shelter, clothing, assisting with birth registration, social workers)	91	78	80	87
Livelihood support (e.g. income generation activities, savings groups)	91	78	70	37
Legal support	9	67	60	25
Nutritional support (incl. referrals)	100	56	60	87
Spiritual and/or emotional support	82	89	80	87
Orphans and vulnerable children care	91	89	100	75
Referral of clients to clinical services	82	100	80	94
Organise/provide transport to health facilities	73	67	60	62
Educate members of the client’s household	82	89	90	94
*Assistance in local health facility*				
Pre-ART counselling	64	78	30	25
HIV pre-test information	73	78	20	19
HIV testing	82	67	10	19
HIV post-test counselling	63	67	20	19
Administrative tasks	9	78	0	19
Other (miscellaneous)	9	78	30	19
*Specific types of support*				
Specific support for women: women and child protection and sexual and reproductive rights interventions	30	56	20	44
Specific support for women: empowerment and self-reliance interventions	10	56	30	6
Lobbying and advocacy activities	20	67	20	31

From their genesis to the present day, CHBC programmes have articulated primary and public health and community welfare in social terms. Large-scale international funding in the mid 2000s, enabled many CSOs to expand their services to address the social needs voiced within communities. To illustrate, an informant from South Africa described the growth of his NGO’s activities in the following terms:

“I mean, we were formed as an HIV organisation, as many were, but we… have been broadening consistently ..… the definition of community health work around non-communicable disease, we’ve expanded our work from prevention, vertical transmission of HIV and breastfeeding……[it now] includes the whole road to health care which is immunisation, developmental stages, child development, … it includes everything now.” Manager of a large care organisation, semi-structured interview^c^.

It should be noted that in Malawi and Zambia in particular, government policies and legislation interlink health, social and economic agenda and emphasise state commitment to community welfare and development. The results also revealed that by 2010/2011 governments’ strategies were beginning to define the future course of CHBC programmes; the emphasis being on promotion of co-ordination of government and civil society initiatives and, with the exception of Ethiopia, an agenda to integrate CHBC into government PHC services as we outline below.

### Co-ordination and integration of CHBC in PHC

Our results included observations in each country on the interactions between CHBC providers, PHC facilities’ staff and state-paid community health workers (CHWs). We present these results below and, recognising that those interactions are influenced greatly by the policy and regulatory contexts in each country, we provide that background in separate text boxes at the end of this article.

The delivery of CHBC services in Malawi is a responsibility of the Ministry of Health (MoH) and is presented as an integral part of the latter’s bio-medically oriented PHC interventions. The role of CSOs and their community care providers is primarily to support state employees who work in health facilities and the state-paid CHWs (the Health Surveillance Assistants, HSAs) in the community. The community care providers operate in a defined geographical area by virtue of being selected by community residents and leaders. They are accountable to the community leadership structures and thus village headpersons and group village head persons play an important supervisory role in the operations of CHBC programmes. CHBC programme staff indicated that the national policy on CHBC, which defines the scope of CHBC and the role of CHBC providers, as well as presence of a framework for collaboration and co-ordination guided their daily activities. The study found (in both the primary research and in the validation questionnaire survey) that volunteer community care providers in Malawi worked alongside the HSAs in PHC activities such as health education, education on water and sanitation and in child immunisation campaigns. Interviews with external stakeholders of CHBC programmes, such as district level hospital staff, and CHBC programme staff also revealed that community care providers fulfil tasks which would normally be provided by a trained community nurse during home visits. This is a deliberate effort of the MoH in which both relatives of patients and community care providers are trained and mentored to provide pressure area care, wound cleaning and physiotherapy. At national level, MoH officials acknowledged the value of community care providers as carers and as a mechanisms that alleviates the burden on PHC facility staff. However, informants from large care organisations expressed their concern with this defrayal of the burden of care and support onto the affected communities. One informant stated that the work at community level relies on volunteers, who according to him,

“are being abused. These are poor people who also need assistance, but they work for free with very little incentives”. Manager of a large care organisation, semi-structured interview.

Data from interviews, FGDs and the community mapping exercises revealed extensive overlapping networks between the different actors involved in PHC service delivery. HBC co-ordinators employed by the MoH are responsible for co-ordinating all district level community care and support activities. This includes ensuring that adequate clinical support is provided to clients and HBC supplies are available for provider and monitoring of HBC provision to ensure quality in service delivery. District co-ordinators of the National AIDS Commission co-ordinate HIV-related activities which include areas such as prevention, treatment, care and impact mitigation. Below this, and under the supervision of PHC facility nurses, HSAs work with community care providers from CHBC programmes, village health and water committees consisting of settlement residents and with patient support groups and are the liaisons for community-based health services. The quote below illustrates.

“There is a very strong link between the primary health care system and community caregivers. This is done through the HSAs and the community nurses. These work directly with both primary and secondary caregivers during periodic home support visits” District HBC co-ordinator, Ministry of Health, semi-structured interview.

Other local-level government employees, such as social welfare assistants, community development assistants, child protection workers and agricultural extension workers, have similar roles as the HSAs with regard to community structures, in relation to their own departments programmes. At community level, they liaise with each other and with the HSAs. The interactions between all these different actors and agencies are guided by 14 national policies and guidelines, including an updated CHBC policy formulated in 2010, and which frame a well-developed PHC system [45]. Notably, the Malawian case demonstrates substantive co-ordination of the activities of an array of different agencies which address both social as well as the bio-medical aspects of health care.

In Zambia, the Ministry of Community Development, Mother and Child Health is responsible for co-ordinating all community-level operations from governmental and non-governmental agencies for health, welfare and community development, including CHBC programmes. This is a recent development, whereby the responsibilities for PHC facility operations were moved from the MoH to this new Ministry in 2013. Officials from the new Ministry as well as the MoH explained that the rationale for this shift was to address the social determinants of health by aligning the governmental departments of primary health, social welfare and community development. Prior to this policy and strategic initiative, in 2012, the MoH completed the training and deployment of the first batch of a projected 5,000 ‘Community Health Assistants’ (CHAs) as part of the country’s PHC revitalisation strategy. These state-paid CHWs are an extension of PHC facility medical teams and their responsibilities were primarily with regard to promotive and preventive health at the community level, rather than curative work, and the scope of their work includes HIV, malaria, respiratory illnesses, diarrheal diseases and tuberculosis. In addition, a campaign was launched in the same year to recruit an additional 3,000 nurses in support of the revitalisation of PHC in the country. At the time of this research, CHAs had not yet been dispatched but they were expected to supervise the activities of CHBC programmes in PHC service delivery. According to national level MoH officials, there are also district-level MoH staff whose job is to co-ordinate PHC facility- and CHBC programmes. Medical staff at health facilities maintain the linkages with CHBC programmes, and with local structures such as neighbourhood health committees, health centre advisory committees and community AIDS task forces.

National-level MoH officials commented that there had never been sound co-ordination of MoH’ PHC services and CHBC. However, discussions with representatives from communities highlighted strong local level networks involving staff from health facilities, the CHBC programmes, and local village/town structures, often involving also local government officials, to facilitate co-ordination of government and non-governmental agencies in the delivery of PHC services. Furthermore, reflecting the changes that were occurring at the time of the research, managers of CHBC programmes noted that they were increasingly being requested to report on their activities to such structures (health centres and local government). Likewise, one national-level MoH official noted:

“The Ministry of Health is interested in the continuum of care of patients from the hospital from, health centre to the community. As you are aware, our vision from the Ministry is to bring these services as close as possible to the family and so we are interested in the care that is given to the patients, even in their home because we do realise that most patients are nursed at home by either relatives or friends so the Ministry of Health defines that community care and support as part of the continuum of care of services”. Director Ministry of Health, semi-structured interview.

In contrast, the researchers recorded that CHBC programme staff had little knowledge at the time about the MoH plans for, and responsibilities of the CHAs. The Zambian case represents a health system in transition with the creation of mechanisms to integrate CHBC programmes into government PHC and socio-economic development services.

In South Africa, in 2010, the national Department of Health (DoH) compiled a new set of policy guidelines as part of their ‘PHC re-engineering strategy’ [49,50]. This strategy aimed at revitalising PHC through state-paid generalist CHWs who would be deployed in outreach teams and supervised by nurses at the PHC facility. Their main roles will be community profiling, risk identification, health promotion and education and referral.

This policy direction implies a change in the current situation whereby non-governmental organisations (the common, formal term is non-profit organisations [NPOs]) are contracted by the DoH to deliver community-level services and through which stipends to community caregivers are being channelled. The new policy proposes that the DoH takes over the ‘employment’ of CHWs from the NPOs. The NPOs will instead be funded for community mobilisation and other community level activities and complement the work of PHC outreach teams. It is further envisaged that these outreach teams will collaborate with the CHWs who fall under the Department of Social Development (DSD) in the provision of psycho-social support. The change in policy is an attempt to address the large variation between CHBC programmes in terms of management of the care workers and scope of work. For example, stipends between programmes tend to differ, depending on whether the programmes receive additional funding such as from PEPFAR or the European Union. A DoH-initiated audit on CHWs revealed that a total of 2,800 NPOs and 72,839 people were providing community-based health services in the country, with no less than nine different categories of CHWs [51]. An official from the DoH explained the need to integrate these roles into one cadre:

“Through re-engineering we thought we don’t need a specialist community health worker specialising in HIV, we need a generalist community health worker in South Africa. I think that is where we are”. Director, National Department of Health, semi-structured interview.

This informant further stated with regard to the variety of NPO programmes, and the need to better co-ordinate their activities, the following:

“Those linkages between community health workers or PHC outreach teams and NGOs will need to be maintained, one form or another. So I still see, for me I still see a role for NPOs, but ....the main, the focus – I think what people need to understand is that our policy direction is ward based, so nobody’s going to work all over – and in view of total partners – that nobody is going to … go into a district and say ‘I have a programme from other funding’ or ‘a programme I want implemented here’ without having looked at district health plans and so on. No it’s not going to happen. We have taken a strong view. So I think for us the ward system as a co-ordinating mechanism … is going to help – we’ll know how many NPO’s in each ward of this country, and even know how many development partners are working about the district in that ward – we have started that process”. Director National Department of Health, semi-structured interview.

A key feature of the DoH initiative is the bio-medical emphasis in its conception of PHC. Interviews with large care organisations and local level CHBC programmes staff revealed that they were well aware of this restriction in terms of stated concerns about how comprehensive PHC services will be in the future and anxieties about whether management of their care and support work by CHWs and PHC facilities would lead to exclusion of their psycho-social care and support services.

In addition, government officials and CHBC programme informants stated that there was no clarity about the future status and role of the DSD CHWs: whether they would be replaced by the new cadres of state (DoH)-paid CHWs or, if not, how the work of these different CHWs would be co-ordinated. Indeed, there were more questions than answers at the time of the research about the actual extent of co-ordination of the different agencies involved in the delivery of PHC services. NPOs, for example, generally recruited their own caregivers without involvement of community representative structures. Our study noted that NPOs referred people to PHC clinics and, in some cases, to DSD welfare services but that there were no evident linkages to other local government departments. The policy and guidelines, as drafted in 2010, were known in all nine provinces but, in 2012/13, there was fragmented implementation. To illustrate, the policy is binding upon the provincial governments but not the implementation guidelines and, reportedly, the Western Cape government had rejected the guidelines, preferring to work according to its own plans for integrating CHBC programmes into PHC services, which include its own procedures for recruitment and payment of CHWs.

The South African case represents a nascent process to integrate CHBC into government PHC services by changing the contractual basis on which the government supports CHBC programmes. The general intention is to replace the existing method of contracting of NPOs to provide community care workers, with state-paid CHWs.

In Ethiopia, the alignment of CHBC programmes with PHC services is complicated because the interactions of the various agencies are largely informal, in a context where the national government has yet to formulate a formal policy on CHBC, where the Federal Ministry of Health (FMoH) regards CHBC programmes as civil society initiatives that have little bearing on its responsibilities to develop PHC services, and where there is now a convoluted process of government restructuring of the governance of the HIV/AIDS programme, PHC services and CHBC. In this instance, it is appropriate to read first the text box on Ethiopia’s policy context prior to reading the results of the research.

Interactions between CHBC programmes, PHC facilities and health ministry officials revolve primarily around the activities and interventions of the FMoH-employed Health Extension Workers (HEWs). According to government officials, HEWs assume a coordinating role by referring patients to both health facilities for clinical care and to CHBC programmes for non-clinical care, and a supervisory role by educating family primary caregivers on how to provide home-based care for patients with HIV. Informants from large care organisations added that HEWs in the city of Addis Ababa, supervise volunteer caregivers from CHBC programmes in the sense that they require the latter to report regularly on their activities to them. In Addis Ababa and, reportedly, some other cities, the HEWs include professional nurses who, it seems, have de facto authority over community volunteers by virtue of their experience, expertise and employment and, therefore, they can persuade these volunteers to assist them.

The ethos of a strict separation between clinical and ‘non-clinical’ aspects of care is endorsed by the re-definition of the HIV/AIDS Prevention and Control Office’s (HAPCO) responsibilities. The clinical components of HIV care are the domain of health care facilities (under supervision of the MoH) while the non-clinical components (i.e. psycho-social and economic support) are the domain of the CSO-led CHBC programmes (under the supervision of HAPCO). However, CHBC programmes do report their activities to the health authorities in their constituencies and CHBC programme staff informants stated that they work closely with local authorities, community groups (idirs), patient associations and other CSOs in the delivery of care and support. The results of the validation questionnaire survey in Ethiopia affirmed this finding. Notably, community volunteer caregivers are selected by representative committees of the community groupings. These agencies work with HEWs to identify people in need of health care and, refer them, as necessary to PHC clinics, to hospitals and to CHBC programme services. Yet, discussions with community volunteers revealed that HEWs have limited involvement in HIV/AIDS-related activities. The HEWs are primarily occupied with issues of sanitation in the community. It is the volunteers who provide counselling, follow up on the adherence of clients on ART and trace ’treatment defaulters’ for the health facilities. Where possible, volunteers train family members of patients on how to provide HBC.

Government officials at national as well as district levels noted that the role of the HAPCO in monitoring and co-ordination of community programmes was not sufficiently executed. The lack of guidelines and functional co-ordination mechanisms for CHBC results, reportedly, in a duplication of efforts and different approaches to care and support within the same district. One informant explained:

“HAPCO is doing capacity building and playing a co-ordinating role. But it is limited. As a result, you see different approaches and activities even the same region, for instance in Amhara region, you see different activities in different district”. Manager of a large care organisation, semi-structured interview.

Current government policy in Ethiopia does not seek to integrate CHBC into PHC services. The collaboration between NGO-run CHBC programmes and government health facilities consists of referral linkages between them, and is guided by a demarcation in clinical and non-clinical care. The quote below illustrates this. The demarcation equally applies to the co-ordination mechanisms between government departments and between them and CSOs.

“Anything done at community level is now decentralised to HEWs. If there is, for instance, an organisation working on care and support in given area, a HEW is responsible to identify needy people, select them on the basis of their problem (social need, economic need) and finally creates linkage to that organisation. They are also working on referral for health issues to nurses. Therefore, community based care and support is about making referral linkage for needy people”. HIV/AIDS advisor, Regional Health Bureau (MoH), semi-structured interview.

### Comparative assessment

The research revealed cross-country similarities in the evolution of CHBC programmes since the introduction of ART. Service provision in all four countries changed from palliative to chronic care, which entailed a diversification of care and support activities in CHBC programmes. Another similarity was observed in the limited government involvement towards CHBC programmes during the pre-ART period. CSOs established programmes independently, which were guided either by international guidelines or self-developed care manuals. The ART programme induced firmer referral linkages between CHBC programmes and health facilities in the four countries but variable institutionalisation of collaboration. South Africa was the first country in which legislation formalised the position of CHBC programmes within the national health system (1997 & 2004). Malawi and Zambia followed in 2005 and 2007 respectively. The main difference was that the South African government contracted CSOs to provide CHBC services whereas in Malawi and Zambia the programmes had been set up independently of government PHC initiatives and governments later sought to regulate them; for example, by introducing common standards for training of caregivers and provision of care. Ethiopia is the exception in that. The federal health ministry regarded them simply as civil society initiatives and the national government did not formulate a policy to foster co-ordination between them and with the health services, let alone integration.

We observed similarities in the way in which the four governments seek to accommodate CHBC programmes. The route is via making PHC facilities the focal point for management and state-paid CHWs responsible for supervising all community health-related activities, including those of CSO-run CHBC programmes. We have noted that this role has not yet been as clearly defined in South Africa and Ethiopia as in Malawi and Zambia whilst in Ethiopia, there is evidence that HEWs are informally taking on this role.

The differences between the countries lie in the relations between government and non-governmental actors and the mechanisms in place to co-ordinate community health activities. South Africa, Malawi and Zambia are cases in which the relations between government and non-governmental actors are guided by legislation and co-ordination mechanisms. In Malawi and Zambia there is some channelling of national funds to CHBC programmes but not for the employment of community volunteers. In both countries, CHBC programmes function on the bases of an ethos of volunteerism and humanitarian assistance by settlement residents. Malawi stands out as a country where the government has actively promoted collaboration with CSOs and between officials of different local government departments and where there is a sophisticated system for co-ordination of different government and non-government agencies. Zambia is following that path. Both countries have PHC frameworks which support the interventions that address the social determinants of health. In Malawi, this is put into practice via co-ordination structures between health and social welfare actors on the ground. In Zambia, there is a Ministerial restructuring in process which aims to align the activities of civil servants in health, welfare and community development. In South Africa and Ethiopia, health and welfare interventions of the various actors at PHC level are separated.

## Discussion

Part of the rationale for an historical, comparative study was its power to provide a context sensitive analysis of how CHBC programmes have evolved and are being addressed in national PHC revitalisation strategies. The research design included a focus on the principles and practice of integration and co-ordination because they are core constructs in the delivery of comprehensive and a continuum of care which, themselves are key principles of PHC.

The complexity of integration and co-ordination within health systems is a frequent topic in literature [41,52-54]. A common understanding in the application of these principles is that ‘one size doesn’t fit all’ and that one can distinguish many forms in practice [55]. Our inter-country assessment confirms this. We could generally distinguish three approaches in the integration of HIV-focussed CHBC in PHC revitalisation strategies: by supervision (Malawi, Zambia), contracting (South Africa) or referral (Ethiopia). They represent the current outcomes of a process in which civil society-led CHBC programmes developed since the 1980s and gained prominence and, over time, government recognition in the delivery of care and support to HIV-infected and affected community members.

This research contextualises changes to the governance of PHC and CHBC services. The findings indicate government reviews of how to integrate and coordinate initiatives, and subsequent adjustments to counter unintended, negative effects (e.g. South Africa; controlling variation) or take it a step further (Zambia; aligning government-led community activities).

Common strengths across the four countries are evident government commitment to revitalise PHC and the strong presence of actors delivering community-based services. CHBC programmes in these countries, in which service delivery cuts across health and non-health domains and began to extend beyond one priority disease, bring a vital resource to the PHC revitalisation agenda. Furthermore, they support establishment of primary care networks which are capable of addressing the health and social care demands of patients with chronic conditions.

As our findings showed, the needs of patients on lifelong ART were as much for medical treatment as for social care and support; which (except from the ART itself) CHBC programmes provided. In the literature on integrated care, care and cure are commonly divided according to the sectors of social welfare and health respectively [36,56,57]. Scholars Kodner and Spreeuwenberg (2002) emphasize that integration essentially is about connectivity, alignment and collaboration within and between the two sectors [58]. This conception is broader than the definitions used in the Ouagadougou declaration, which refer to the health sector. Current policy direction in South Africa reflects the latter (which is a shift away from earlier policy frameworks which incorporate the social welfare sector), as do the recent developments in Ethiopia. The Malawi case study demonstrates the presence of a network in which the two sectors are connected via substantive structures and co-ordination mechanisms which link services and staff from the different government departments (with clearly defined responsibilities) with CSOs, community authorities and residents. Zambia is in the process of emulating this approach.

Experiences with aligning the health and social welfare sectors in high-income countries have shown variable success [59-62] but a necessary ongoing process in view of changing health care demands as a result of ageing populations and chronic illnesses [63,64]. Our research shows that out of four, two countries are already directing their PHC revitalisation efforts towards this goal.

This study focused on programmes which provide care and support to HIV patients in particular. This influences the representativeness of the study findings, for there are other community programmes (e.g. care for the elderly; for orphans) which were not covered by this study. It also does not do full justice to the broad scope of community care and support programmes available in the four countries. However, the changes within HIV-focused programmes following the introduction of ART reiterate the need for addressing the social determinants of health in a community, and as such provided an interesting angle to explore PHC revitalisation strategies. A strength of this study is the use of different data sources in the construction of four country cases. The historical perspective has allowed for further contextualisation and for the illustration of novel approaches in care.

## Conclusion

Our sample brought together four countries which share an evident government commitment to revitalise PHC and a strong presence of actors delivering community-based services. We described the overlap between the two, according to the principles of integration and co-ordination. In our assessment of how governments were seeking to incorporate CHBC programmes as part of their PHC revitalisation strategies, we distinguished three approaches: by supervision (Malawi, Zambia), contracting (South Africa) or referral (Ethiopia). We also observed contextual differences in the relations between government and non-governmental actors and in the mechanisms for co-ordinating community health and social welfare activities.

In all countries, CHBC programmes diversified their services, following the introduction of ART. We observed a trend amongst CHBC providers to extend their services beyond HIV-infected patients. With this, CHBC programmes in Ethiopia, Malawi, South Africa and Zambia bring a vital resource to the public delivery of primary health and social welfare services. In a context of changing demands on the health system, this expanded capacity in integrated care will be an important necessity for the future. A process of continuous reflection on the course of the PHC revitalisation agenda, as well as directed investment in community capacity, will help augment the results of current efforts in each country.

### Background information on policy and regulatory contexts per country

#### Malawi

Malawi represents a PHC system which reflects the aims of the revitalisation agenda but which preceded the Ouagadougou declaration of 2008. In 2004, the government outlined the core PHC interventions provided to the community in an essential health package [65]. The Ministry of Health (MoH) deploys state-paid Health Surveillance Assistants (HSAs) whose responsibilities are mainly in surveillance, support to immunisation campaigns, growth monitoring and treatment of minor illnesses. HSAs have been employed in Malawi since the 1960s though in those days they were known as ‘small pox vaccinators’ and ‘cholera assistants’ [66]. Their roles have expanded over time to incorporate collection of sputum samples from TB patients, recruitment of HIV patients into the national ART programme, providing support to, and supervision of CHBC programmes in their designated operational areas, promoting use of insecticide-treated mosquito nets and safe birth delivery practices. There are also HSA-equivalent employees from other ministries whose responsibilities are to monitor and facilitate local-level government projects and who liaise with each other; for example, Social Welfare assistants and Community Development assistants.

CHBC has long existed in Malawi but was not formally recognised because caregiving is considered a cultural responsibility. CSOs operated independently and chose the location and nature of their services. In 1993, the National AIDS Commission started to co-ordinate and fund CSOs to undertake community-based interventions including care and support, as part of the national strategic framework. In 2005, further co-ordination and standardisation of CHBC programmes was pursued by government when it issued a national policy on CHBC and another on palliative care. In 2011, the CHBC policy was updated to incorporate a wider range of beneficiaries, including those living with chronic conditions [67]. The policy defines the scope of practice for actors involved in CHBC. These include trained health workers, HSAs, support groups, community volunteers (in Malawi referred to as community care providers) as well as staff from other ministries such as Social Welfare.

#### Zambia

The CSO-founded CHBC system has been accepted as a vital component of PHC in Zambia for many years. That recognition stems from the mid-1980s when the government introduced decentralised health care in accord with the 1978 global commitment to developing PHC services. Decentralisation involved the establishment of ‘health centres’ and ‘health posts’ in settlements and, in 1991, ’health advisory committees’ and ‘neighbourhood committees’ to enhance community involvement. At the same time, parish clergy and Christian mission hospitals were mobilising communities on humanitarian grounds to assist HIV/AIDS patients. The Salvation Army, for example, pioneered use of mobile medical health teams to provide home-based palliative care. In time, this HBC model came to rely upon community volunteers due to the high costs of running the mobile units. CSO HBC initiatives worked with, and supported the PHC facilities but were HIV-focussed and independently organised. From a hierarchical perspective, CHBC programme personnel constituted the lowest level of PHC personnel but, from an organisational perspective, they were a parallel workforce to the health ministry PHC staff and other local government employees. That situation continued throughout the 1990s, during which international donors funded the growth of CSO initiatives and the government instituted standards for HBC.

The mid-late 2000s witnessed government attempts to ‘formalise’ the place and role of CHBC programmes within the national health system [68]. In 2004, the MoH gained the authority to manage both the national HIV/AIDS response and direction of the health system. Previously, that authority lay with the Central Board of Health which co-ordinated sectoral activities and allocated funding but which was disbanded in 2004, the time when Zambia began to receive large inflows of international funding for its ART programme [69,70]. Thereafter, the MoH focused on its mandate: setting up the public ART programme, developing minimum standards for community- and home based care organisations in 2007 [68] and, since 2008/9, seeking to create an integrated PHC service structure. The current ‘National Health Strategic Plan, 2011-2015’ [71] and ‘National Health Policy’ [72] emphasise government commitment to revitalise PHC. In 2013, government restructured its Minstries and created the Ministry of Community Development, Mother and Child Health in which it aligned PHC interventions with those of social welfare and community development.

#### South Africa

Before 1994, community health workers played an important role in complementing government health services to redress the effects of the inequitable health service delivery during apartheid. In the late 1990s, community health services delivered by CSOs increased in response to the HIV epidemic. Legislation, the Non-Profit Organisation Act of 1997, enabled these programmes to run with the DoH defining the need and service standards for CHWs, identified as community-based volunteer ‘careworkers’. The DoH also introduced guidelines for the payment of stipends to these CHWs and both the DoH and the Department of Social Development (DSD) started the provision of grants to CSOs to establish HBC programmes. The departments set their own rates for stipends to be paid to careworkers. Though many of these HBC programmes evolved into CHBC programmes on the basis of volunteer careworkers, subliminally, there has always been a financial contractual premise in their recruitment and deployment as a result of the payment of stipends (i.e. the volunteers regarded care work as a job, albeit poorly paid). Careworkers are commonly recruited, selected, supervised and paid by NPOs themselves, who are obliged to report to the DoH or DSD at district level. At provincial level, NPOs are co-ordinated by HBC programme coordinators.

In 2004, the DoH drafted a CHW policy framework. This framework was updated to the ‘Community care worker policy management framework’ in 2009 by the DoH and the DSD. The update was deemed necessary as the growth in numbers of lay workers and different management models across the funded NPOs had caused considerable variation in e.g. the conditions of service among community care workers. The 2004 and 2009 frameworks were never formalised. Following extensive consultations between the DoH and CSOs to define the role and place of CHBC programmes within the public health system, a new policy was drafted in 2010 [49]. The DoH policy is to create cadres of state-paid CHWs to work as an extended arm of PHC health care teams. The policy defines PHC as a biomedical construct, in which care and support roles as currently taken up by psychosocial- and CHBC programmes are not included.

#### Ethiopia

In 2003–2004, the Ethiopian government launched the Health Extension Program to expand PHC services. The program has included efforts to upgrade or, as necessary, construct 15,000 health posts and 3,200 health centres and to recruit 30,000 female health extension workers (HEWs) from local communities, who receive one year of training before being assigned (in pairs) to run ‘health posts’ and to work with families [73,74]. Their responsibilities are hygiene and environmental sanitation, family health, disease prevention and control, and health education and communication.

CSOs established HBC and CHBC programs independently of government’s interventions and before the government formulated a national HIV/AIDS policy in 1998 [75]. The policy allocated the responsibility for establishing and managing a vertical HIV/AIDS program to the Federal Ministry of Health (FMoH), via its own ‘HIV/AIDS Prevention Control Office’ (HAPCO). The FMoH acknowledged CSO initiatives as informal social welfare services separate to the country’s health services and, in principle, as the responsibility of the Departments of Finance and Economic Development and of Labour and Social Affairs. Informally, there was collaboration between CHBC programmes and district-level HAPCO officials and health facilities such that the former were involved in supporting PHC services.

The 1998 policy was still in force in 2012 but the government had begun, in 2011, to initiate structural changes for the treatment, care and support of HIV/AIDS patients via a national task force managed initially by the FMoH and, later, by the Ministry of Women Children and Youth Affairs. The HAPCO was redefined as the agency responsible for co-ordinating CHBC programmes and to take responsibility for community sensitisation, resource mobilisation and the multi-sectoral response to HIV/AIDS. CSOs are required to sign a memorandum of understanding with the Regional Health Bureau, HAPCO and the Economic and Finance Office before they implement of their programs. The health offices at district level are responsible for monitoring the clinical aspects of HBC provision and the HAPCO for the non-clinical aspects.

To date, there is no government policy or guideline on CHBC in Ethiopia, but informants mentioned that the HAPCO at the Federal level, in collaboration with its funding partners, is preparing a national guideline for palliative care which is inclusive of CHBC service delivery.

## Endnotes

^a^ART patients who are stable on and adherent to their treatment can access their drugs through supervised ‘clubs’ and herewith avoid queuing at ART clinics and pharmacies.

^b^Community members with different expertise and backgrounds form, in effect, mini-multi disciplinary teams which provide diverse care and support to patients, ranging from basic nursing care from those trained in community health, food support from those who have a farming background and advice on income generation from those who are business men/women.

^c^All quotes in this paper are derived directly from transcripts or preliminary interim research reports from the four countries.

## Abbreviations

ART: Anti-retroviral treatment
CBO: Community based organisation
CHA: Community health assistant
CHBC: Community home based care
CHW: Community health worker
CSO: Civil society organisation
DoH: Department of health
DSD: Department of social development
DSW: Department of social welfare
FBO: Faith based organisation
FMoH: Federal ministry of health
HAPCO: HIV/AIDS prevention and control office
HBC: Home based care
HEW: Health extension worker
HIV: Human immuno-deficiency virus
HSA: Health surveillance assistant
MoH: Ministry of health
NCD: Non-communicable diseases
NGO: Non-governmental organisation
PHC: Primary health care
WHO: World health organisation
